# Measurement Equivalence of Diabetes Self-Management, Distress, and Quality-of-Life Measures in Adults with Type 2 Diabetes in Vietnam

**DOI:** 10.3390/nursrep16060205

**Published:** 2026-06-18

**Authors:** Thu-Thuy Thi Nguyen, Huu Thuan Vo, Thi Tuong Vi Nguyen, Pham Minh Son, Vu Thi Xim, Thi My Nhung Pham, Mieu An Phan, Thi Anh Nguyen

**Affiliations:** 1 Faculty of Nursing-Midwifery, Hong Bang International University, Ho Chi Minh City 70000, Vietnam; thuyntt2@hiu.vn (T.-T.T.N.); vintt3@hiu.vn (T.T.V.N.); nhungptm@hiu.vn (T.M.N.P.); anpm@hiu.vn (M.A.P.); 2 Faculty of Nursing, Nguyen Tat Thanh University, Ho Chi Minh City 70000, Vietnam; thuanvh@ntt.edu.vn (H.T.V.); sonpm@ntt.edu.vn (P.M.S.); vtxim@ntt.edu.vn (V.T.X.); 3 Cho Ray Hospital, Ho Chi Minh City 70000, Vietnam

**Keywords:** measurement equivalence, type 2 diabetes, diabetes distress, self-management, quality of life, low- and middle-income countries, educational attainment, multi-group confirmatory factor analysis, nursing

## Abstract

**Background:** Patient-reported outcome comparisons require measurement equivalence, which is seldom tested in low- and middle-income country (LMIC) diabetes research. We examined equivalence of the Diabetes Self-Management Instrument-35 (DSMI-35), Diabetes Distress Scale-17 (DDS-17), and Asian Diabetes Quality of Life (AsianDQOL) scale across sex, fasting-glucose stratum, and educational attainment in Vietnamese adults with type 2 diabetes. **Methods:** We conducted a secondary analysis of 374 adults (female 152, male 222; lower-FBG < 154 mg/dL, *n* = 212; higher-FBG *n* = 162; secondary-or-lower *n* = 202; tertiary-or-higher *n* = 172). Multi-group CFA (lavaan) tested configural, metric, and scalar equivalence of a parcel-level three-factor model (parcel-level equivalence does not imply item-level equivalence). Path equality was evaluated with scaled Satorra–Bentler likelihood-ratio tests; indirect effects were bootstrapped (*n* = 5000). **Results:** Scalar-equivalence change-index criteria (ΔCFI ≤ 0.010; ΔRMSEA ≤ 0.015) were met for all groupings; however, for fasting glucose the configural baseline fit was weak (RMSEA 0.117–0.119), so fasting-glucose equivalence is reported only as provisional and is not interpreted at the level of the sex and education findings. McDonald’s ω was ≥ 0.959 in every subgroup. Structural paths did not differ by sex (Δχ^2^(3) = 1.18, *p* = 0.758; not powered for equivalence) but differed by education (Δχ^2^(3) = 71.16, *p* < 0.001), with the cross-sectional association structure differing by education (distress-channelled in tertiary-or-higher and partly direct in secondary-or-lower participants); because the data are cross-sectional, these are differences in association structure, not established mediation. The fasting-glucose structural comparison was not interpretable because the lower-FBG subgroup (FBG < 154 mg/dL, *n* = 212) had a non-positive-definite latent covariance matrix. **Conclusions:** Scalar equivalence criteria were met for sex and education and only preliminarily supported for fasting-glucose stratum, where elevated configural RMSEA (0.119) cautions against firm interpretation. The self-management → distress → quality-of-life pathway showed no detected sex difference but differed by educational attainment. Measurement equivalence testing, including configural-fit assessment, should be routine in LMIC patient-reported outcome validation.

## 1. Introduction

Type 2 diabetes mellitus (T2DM) affects an estimated 537 million adults globally, approximately 80% of whom live in low- and middle-income countries (LMICs) [1,2]. In the Western Pacific region, adult T2DM prevalence is projected to reach 260 million by 2045, including 151 million in Southeast Asia [2]. Psychosocial burden, including poorer self-management, higher diabetes distress, and lower health-related quality of life (HRQoL), varies across sex, glycemic status, and educational attainment [3,4]. Identifying these subgroup patterns is essential for targeted, resource-efficient interventions in capacity-constrained health systems.

Valid subgroup comparison requires measurement equivalence: evidence that a scale measures the same latent construct with comparable precision across groups [5]. We use measurement equivalence and measurement invariance synonymously, consistent with psychometric usage [5,6,7]. Without equivalence, apparent subgroup differences may reflect differential item functioning rather than true construct differences. Multi-group confirmatory factor analysis (MG-CFA) commonly evaluates configural, metric, and scalar equivalence [6]. Scalar equivalence is the minimum requirement for comparing latent means; without it, subgroup score comparisons are methodologically unstable.

Despite frequent subgroup comparisons in T2DM research, measurement equivalence is rarely tested in LMIC settings. Diabetes-specific patient-reported outcomes (PROs) are often translated and validated in full Asian samples [8,9,10], but full-sample validation does not establish equivalence across sex or glycemic strata. To our knowledge, no published Southeast Asian or LMIC T2DM study has tested measurement equivalence of the DSMI-35, DDS-17, and AsianDQOL across sex, fasting-glucose stratum, or educational attainment; broader claims about diabetes patient-reported-outcome research generally are beyond what our literature search can support. This leaves a major validity assumption unexamined.

The DSMI-35, DDS-17, and AsianDQOL are relevant to Vietnamese and Southeast Asian T2DM research. The DSMI-35, validated for Vietnamese T2DM by Dao-Tran et al. [11], assesses five self-management domains. The DDS-17, developed by Polonsky et al. [12] and adapted into Vietnamese by Thinh et al. [13], measures diabetes-specific emotional burden. The AsianDQOL captures HRQoL domains relevant to Asian T2DM populations [14]. Although each has acceptable psychometric evidence in Vietnamese or Asian samples [11,13,14], their subgroup equivalence has not been established.

We examined measurement equivalence of the DSMI-35, DDS-17, and AsianDQOL across sex, fasting-glucose stratum, and educational attainment in 374 Vietnamese adults with T2DM. Using MG-CFA, we tested configural, metric, and scalar equivalence using ΔCFI ≤ 0.010 and ΔRMSEA ≤ 0.015 criteria [6,15]. Secondarily, we compared self-management → distress → quality-of-life structural paths across groups where scalar equivalence was supported.

## 2. Methods

### 2.1. Study Design and Participants

This secondary analysis used cross-sectional data from 374 adults with T2DM recruited from two private hospitals in Binh Duong Province, Vietnam, between June and July 2024. Eligibility criteria were confirmed T2DM diagnosis, age ≥ 18 years, and ability to complete self-report questionnaires. Data collection and this secondary analysis were approved under TUA-IERC Protocol 2024-033-Nguyen-CNU-Diabetes-v2 (24 June 2024).

### 2.2. Instruments

DSMI-35. The Diabetes Self-Management Instrument-35 measures five self-management subscales: self-regulation (10 items), self-integration (9 items), self-interaction (9 items), self-monitoring (4 items), and medication adherence (3 items). Items are rated from 1 (never) to 4 (always). The Vietnamese version was validated by Dao-Tran et al. [11].

DDS-17. The Diabetes Distress Scale-17 assesses four domains: emotional burden (5 items), physician-related distress (4 items), regimen distress (5 items), and interpersonal distress (3 items). Items are rated on a 6-point scale. The Vietnamese version was adapted by Thinh et al. [13].

AsianDQOL. The Asian Diabetes Quality of Life scale measures diet (6 items), energy (3 items), memory/cognition (4 items), financial burden (5 items), and interpersonal relations (3 items) on a 4-point scale. It was validated by Goh et al. [14] and forward-translated into Vietnamese by two bilingual diabetes-nursing experts and administered after a 30-participant pilot; formal back-translation, expert-committee reconciliation, and cognitive debriefing were not undertaken.

### 2.3. Grouping Variables

We examined sex, fasting-glucose stratum, and educational attainment. Sex was coded as female (*n* = 152) or male (*n* = 222). Fasting-glucose stratum used a prespecified fasting blood glucose (FBG) cut point at 154 mg/dL: lower-FBG (FBG < 154 mg/dL, *n* = 212) and higher-FBG (FBG ≥ 154 mg/dL, *n* = 162). We use lower/higher rather than controlled/uncontrolled because clinical glycemic control is conventionally indexed by HbA1c, often with a target < 7.0% for many non-pregnant adults [16]. The 154 mg/dL cut point is the estimated average glucose (eAG) equivalent of an HbA1c of 7.0% (eAG [mg/dL] = 28.7 × HbA1c − 46.7, giving 154 mg/dL at 7.0%) [17], J; because HbA1c was not recorded in this dataset, FBG was used as a proxy stratifier anchored to this clinically meaningful value, and it also fell close to the sample median FBG (149 mg/dL). A single FBG value does not establish control status. This threshold is therefore a sample-stratification device used to mirror common LMIC T2DM PRO comparisons where HbA1c is not routinely available. Educational attainment was dichotomized as secondary-or-lower (*n* = 202) or tertiary-or-higher (*n* = 172) and prespecified as a secondary grouping variable. All groupings met the recommended minimum cell size of *n* ≥ 100 for stable multi-group structural equation modeling. Parcel-level data were complete for all 374 participants.

### 2.4. Statistical Analysis

Subscale means (DSMI-35, DDS-17) and subscale sums (AsianDQOL) were used as parcel-level indicators because within-subscale homogeneity supports parcel representation in samples of this size [18]. Each instrument was modeled as a single latent factor, which departs from the developer-intended multidimensional structures (DSMI-35 five subscales, DDS-17 four domains, AsianDQOL five domains). Thus, the analysis tests equivalence of unidimensional composites, not developer-intended factor structures; subscale- and item-level equivalence remain untested. AsianDQOL sums preserved the original scoring protocol [14]. Internal consistency was assessed with McDonald’s ω using the psych package. MLR estimation was selected after examining skewness and kurtosis. Structural paths are reported as unstandardized B with model-based 95% confidence intervals and standardized beta for effect-size interpretation. Indirect effects use unstandardized percentile bootstrap confidence intervals.

Measurement equivalence was tested with MG-CFA in R (lavaan 0.6–21; [19]). After a common full-sample baseline CFA using MLR, each grouping followed a configural, metric, and scalar sequence. Robust CFI and RMSEA were used for model fit, and equivalence was supported by ΔCFI ≤ 0.010 and ΔRMSEA ≤ 0.015 [6,15]. Modification indices were to be inspected if scalar equivalence failed.

Structural path equality was tested with scaled Satorra–Bentler likelihood-ratio tests comparing free and constrained structural-path models. Bootstrap percentile confidence intervals (*n* = 5000; set.seed = 2024; ML estimator) were computed for subgroup indirect effects. Heavy right skew was observed in tertiary-or-higher (SE = 28.72; 95% CI half-width/SE = 0.075; skewness = 19.2) and female (SE = 9.89; ratio = 0.078; skewness = 70.5) bootstrap distributions, whereas male (ratio = 1.91; skewness = 1.94) and secondary-or-lower (ratio = 1.83; skewness = 2.02) distributions were approximately symmetric. We therefore used percentile CIs rather than Wald z-tests for indirect effects and report diagnostics in Supplementary Table S1. BCa sensitivity intervals also excluded zero for all four displayed subgroup indirect effects. Parcel rules, the FBG cut point, ΔCFI/ΔRMSEA thresholds [6,15], and bootstrap replication count were prespecified. Analyses used R 4.5.x; figures were exported at 600 dpi (TIFF/LZW) and as PDFs.

## 3. Results

### 3.1. Sample Characteristics

The sample included 374 adults with T2DM: 152 female (40.6%) and 222 male (59.4%). Mean age was 53.0 ± 2.9 years for females and 52.8 ± 2.8 years for males. Mean BMI was 22.9 ± 2.2 and 23.0 ± 2.1 kg/m^2^, respectively; mean FBG was 150.1 ± 24.9 and 149.0 ± 24.3 mg/dL. Mean diabetes duration was 8.5 ± 3.0 years in females and 8.4 ± 2.9 years in males. Overall, 212 participants (56.7%) were in the lower-FBG stratum. Full descriptives are shown in Table 1.

### 3.2. Baseline CFA and Reliability

The full-sample three-factor CFA (*n* = 374) showed borderline acceptable fit: CFI = 0.973, TLI = 0.967, RMSEA = 0.085 (90% CI 0.074-0.096), SRMR = 0.025, scaled χ^2^(74) = 272.20, *p* < 0.001. All standardized loadings were significant (*p* < 0.001). Across sex subgroups, standardized loadings ranged from 0.581 to 0.904 (DSM_lat), 0.962 to 0.978 (DDS_lat), and 0.890 to 0.968 (QOL_lat). McDonald’s ω was ≥0.959 for every instrument and subgroup (DSMI-35: 0.959–0.972; DDS-17: 0.982–0.990; AsianDQOL: 0.970–0.990), indicating excellent internal consistency (Table 2). Although uniformly high, the largest ω values (DDS-17 and AsianDQOL, up to 0.990) may also reflect item redundancy in these multi-item instruments, with implications for respondent burden and scale efficiency; shortened forms warrant future examination. The full-sample baseline RMSEA (0.085, 90% CI 0.074–0.096) modestly exceeds the conventional 0.08 threshold, so absolute fit is interpreted with corresponding caution.

### 3.3. Measurement Equivalence

**Figure 1:**
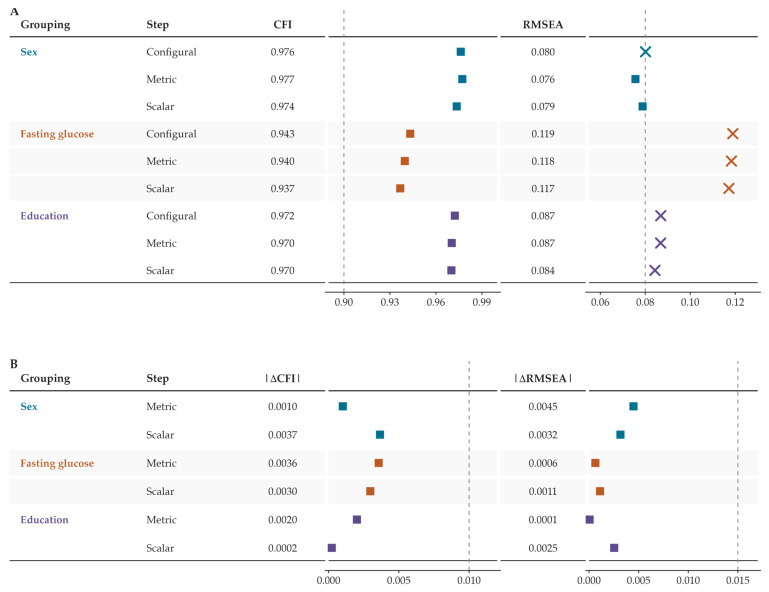
Measurement equivalence diagnostics for the parcel-level three-factor model (DSMI-35, DDS-17, AsianDQOL) across sex, fasting glucose stratum, and educational attainment (*n* = 374). Panel (**A**): Configural, metric, and scalar model fit by grouping. Panel (**B**): Absolute ΔCFI and ΔRMSEA after imposing metric and scalar constraints. Dashed lines mark prespecified thresholds (CFI ≥ 0.90; RMSEA ≤ 0.08; ΔCFI ≤ 0.010; ΔRMSEA ≤ 0.015). Filled symbols meet the threshold; × symbols do not. Threshold comparisons use the unrounded fit-index value; for the sex configural model, the unrounded RMSEA (0.0801) marginally exceeds the 0.08 threshold and is displayed as 0.080. Colors denote the grouping variable: teal, sex; orange, fasting glucose stratum; purple, educational attainment.

Table 3 and Figure 1 summarize all equivalence tests. Figure 1A shows absolute fit (CFI and RMSEA) across the sequence; Figure 1B shows absolute ΔCFI and ΔRMSEA against Cheung and Rensvold [6] and Chen [15] thresholds.

Sex. The configural model had acceptable CFI (0.976) and borderline RMSEA (unrounded 0.0801; displayed 0.080), with the 90% RMSEA CI bracketing 0.08. Metric (ΔCFI = −0.001, ΔRMSEA = −0.004) and scalar (ΔCFI = 0.004, ΔRMSEA = 0.003) equivalence criteria were met, supporting equivalent loadings and intercepts across female and male groups.

Fasting glucose stratum. The configural model showed acceptable CFI (0.943) but elevated RMSEA (0.119). Metric (ΔCFI = 0.004, ΔRMSEA = −0.001) and scalar (ΔCFI = 0.003, ΔRMSEA = −0.001) change criteria were met, supporting equivalent loadings and intercepts by change-index criteria. Because absolute baseline fit was weak (RMSEA 0.119), fasting-glucose equivalence is reported only as provisional and is not interpreted at the level of the sex and education findings.

Education. Scalar equivalence criteria were met across educational attainment groups (metric: ΔCFI = 0.002, ΔRMSEA < 0.001; scalar: ΔCFI = 0.0002, ΔRMSEA = −0.003).

No equivalence test exceeded the ΔCFI ≤ 0.010 or ΔRMSEA ≤ 0.015 thresholds (Figure 1B). However, absolute fit differed by grouping: sex remained within conventional bounds, education had acceptable CFI but RMSEA marginally above 0.08 (0.084–0.087), and fasting glucose had acceptable CFI but RMSEA clearly above 0.08 (0.117–0.119). Thus, change-based criteria supported scalar equivalence across all groupings, but fasting-glucose findings require confirmation in larger samples with acceptable configural fit.

### 3.4. Structural Path Comparison (Secondary)

Sex. With metric equivalence established, structural paths did not differ significantly by sex (scaled Δχ^2^(3) = 1.179, *p* = 0.758). In both groups, DSM_lat → DDS_lat, DDS_lat → QOL_lat, and the direct self-management → quality-of-life path were significant at *p* < 0.01 (female direct β_std = 0.360; male β_std = 0.484), indicating partial mediation. Bootstrap percentile CIs for indirect effects excluded zero in both sexes (female 95% CI [0.199, 1.746]; male 95% CI [0.343, 1.795]) and overlapped substantially, consistent with the LRT. Full estimates are in Table 4.

**Figure 2:**
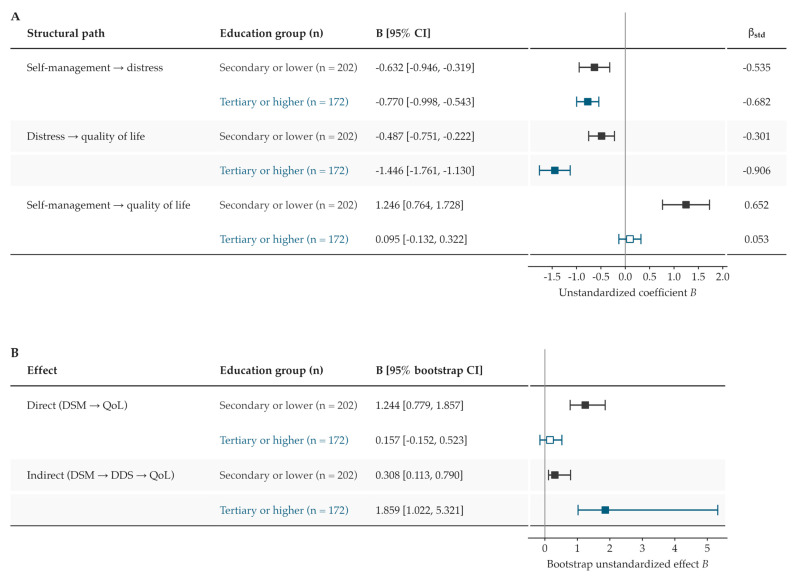
Education-stratified structural paths and mediated effects (secondary-or-lower *n* = 202; tertiary-or-higher *n* = 172). Panel (**A**): Unstandardized structural path coefficients with model-based 95% CIs; β_std shown in the rightmost column. Panel (**B**): Direct and indirect effects with percentile bootstrap 95% CIs (*n* = 5000 replications). Filled symbols indicate intervals excluding zero. Path equality across education groups was rejected (scaled Δχ^2^(3) = 71.16, *p* < 0.001).

Education. Structural paths differed by educational attainment (scaled Δχ^2^(3) = 71.162, *p* < 0.001; Figure 2A). Among secondary-or-lower participants, both the indirect effect (95% CI [0.113, 0.790]) and direct DSM_lat → QOL_lat path (β_std = 0.652, *p* < 0.001) were significant, indicating a partly direct cross-sectional association structure. Among tertiary-or-higher participants, the indirect effect was larger (95% CI [1.022, 5.321]) and the direct path was not detected (β_std = 0.053, *p* = 0.411), indicating a predominantly distress-channelled association structure (Figure 2B). Because the data are cross-sectional, these are differences in association structure, not established mediation mechanisms.

Fasting glucose stratum. Structural path comparison was not interpretable because the lower-FBG subgroup (FBG < 154 mg/dL, *n* = 212) produced a non-positive-definite latent covariance matrix. The estimated distress–quality-of-life latent correlation reached the −1.0 boundary in this subgroup (Supplementary Table S1E), indicating local non-identification (empirical near-collinearity of the two latent factors) rather than insufficient sample size; this stratum was in fact the larger of the two. A valid two-group structural comparison requires both strata to be identified; because the lower-FBG model is not, the fasting-glucose structural comparison cannot be interpreted and would require a respecified latent covariance structure or independent replication.

## 4. Discussion

### 4.1. Main Finding

Subgroup comparisons of diabetes PROs in LMIC settings assume that translated instruments behave equivalently across routinely compared groups. This study tested that assumption for three Vietnamese T2DM instruments (DSMI-35, DDS-17, AsianDQOL). Parcel-level scalar equivalence was supported for sex and education; fasting-glucose-stratum equivalence was inconclusive because configural fit was poor. These findings support parcel-level subgroup comparisons with these instruments in similar Vietnamese T2DM, samples, while underscoring that results do not automatically extend to other tools or to item-level functioning.

### 4.2. Implications for LMIC Research

The lack of measurement-equivalence testing is a systemic gap in LMIC diabetes PRO research. Translated instruments are often used for sex and glycemic subgroup comparisons without MG-CFA evidence that scale properties transfer across groups. If equivalence fails, observed group differences may reflect measurement bias rather than true construct differences. Our findings therefore support subgroup comparisons using these instruments in similar Vietnamese T2DM populations, but only at the parcel level and pending item-level and external replication. Future LMIC validation studies should include equivalence testing as a standard step.

### 4.3. Sex: No Detected Structural-Path Difference

After measurement equivalence was established, the self-management → distress → quality-of-life pathway did not differ significantly by sex (scaled Δχ^2^(3) = 1.179, *p* = 0.758). Standardized paths were similar for DSM_lat → DDS_lat (female β_std = −0.602; male β_std = −0.616), DDS_lat → QOL_lat (female β_std = −0.601; male β_std = −0.467), and DSM_lat → QOL_lat (female β_std = 0.360; male β_std = 0.484). Both sexes showed indirect effects with bootstrap CIs excluding zero (female 95% CI [0.199, 1.746]; male 95% CI [0.343, 1.795]) and detectable direct paths, consistent with partial mediation. This non-difference should be interpreted as absence of evidence for a sex difference, not evidence of equivalence; the analysis was not powered to establish structural-path equivalence, and formal equivalence testing was not prespecified.

### 4.4. Education and the Cross-Sectional Association Structure

Although measurement equivalence was established across education groups, the cross-sectional association structure differed markedly by education (scaled Δχ^2^(3) = 71.162, *p* < 0.001). Among secondary-or-lower participants, both indirect (95% CI [0.113, 0.790]) and direct self-management → QOL associations (β_std = 0.652, *p* < 0.001) were present, indicating a partly direct structure; among tertiary-or-higher participants, the larger indirect effect (95% CI [1.022, 5.321]) and non-detected direct path (β_std = 0.053, *p* = 0.411) indicated a predominantly distress-channelled structure. Because the data are cross-sectional, these are differences in association structure, not established mediation mechanisms.

This education-related difference in association structure may have several population-level explanations. Health literacy is strongly associated with diabetes self-management and tends to be higher with more education [17], potentially channelling self-management benefits through reduced distress, whereas lower health literacy may accompany a more direct, less distress-mediated association [20]. Differences in self-regulation capacity and in access to health information may further shape these associations across education strata; these remain hypotheses to be tested, not established mechanisms. In secondary-or-lower participants, self-management was associated with quality of life through both distress-mediated and direct pathways, whereas in tertiary-or-higher participants the association was mainly distress-mediated. Because these cross-sectional findings are exploratory, prospective studies should test whether they translate into differential intervention response. The bootstrap sensitivity analysis (Supplementary File S2) supports the zero-exclusion inference for these indirect effects, but the heavy right tail in the tertiary-or-higher and female distributions means the point magnitudes should be read as approximate.

### 4.5. Fasting Glucose Stratum: Measurement Properties but Not Structural Interpretability

Change-index criteria for scalar equivalence were met across lower-FBG and higher-FBG strata, but the finding should remain preliminary. The fasting-glucose configural model fit was weaker than for sex or education (CFI = 0.943, RMSEA = 0.119), and RMSEA exceeded the conventional 0.08 threshold. Because subsequent ΔCFI and ΔRMSEA values are evaluated relative to this weak baseline, the change-index pass should be interpreted as a measurement-warning signal rather than confirmatory evidence of construct-level equivalence [7]. The structural model was also not interpretable, owing to a non-positive-definite latent covariance matrix in the lower-FBG subgroup (distress–quality-of-life latent correlation at the −1.0 boundary; Supplementary Table S1E). Replication in a larger sample with acceptable configural fit is required before firm fasting-glucose equivalence or structural-path claims can be made.

### 4.6. Comparison with Existing Measurement Equivalence Literature

Measurement equivalence studies of diabetes-specific PROs remain sparse. To our knowledge, no published study has examined MG-CFA equivalence of the DSMI-35, DDS-17, or AsianDQOL in Southeast Asian or LMIC patient subgroups. Full-sample validation without subgroup equivalence testing leaves comparative findings dependent on an assumption. This study provides initial evidence that the assumption holds at the parcel level for these instruments in a Vietnamese T2DM sample.

### 4.7. Limitations

Several limitations warrant emphasis. First, this cross-sectional secondary analysis used dissertation data from two private hospitals in one Vietnamese province, limiting generalizability. The sample was relatively highly educated (tertiary-or-higher 46%, 172/374), so the education-moderated mediation result should be read as an internal contrast, not a population estimate. Second, fasting-glucose strata were based on one FBG cut point (154 mg/dL), not HbA1c; a single FBG value does not establish clinical control. Third, the fasting-glucose structural model was not interpretable because the lower-FBG subgroup produced a non-positive-definite latent covariance matrix (near-collinear distress and quality-of-life latent factors), leaving fasting-glucose structural-path equivalence unresolved. Fourth, the education grouping was coarse. Fifth, we tested parcel-level composite constructs, not the developer-intended multidimensional item structures; parcel-level equivalence does not establish item- or subscale-level equivalence, and item-level differential-item-functioning analyses (e.g., ordinal logistic DIF or item-response-theory approaches) are a necessary next step. Sixth, the Vietnamese AsianDQOL was prepared by forward translation only, without formal back-translation, expert-committee reconciliation, or cognitive debriefing, which may affect the stability of the latent quality-of-life construct.

### 4.8. Future Directions

Larger multi-site and multi-country LMIC studies should test whether these measurement and structural findings generalize. Longitudinal designs are needed to assess whether the education-stratified associations are stable over time. Item-level equivalence testing could identify specific items that function differently across subgroups and guide instrument refinement. Future studies should extend equivalence testing to other diabetes PROs used in the region.

## 5. Conclusions

The parcel-level three-factor model representing the DSMI-35, DDS-17, and AsianDQOL met scalar equivalence criteria across sex, fasting-glucose stratum, and educational attainment in Vietnamese adults with type 2 diabetes, although fasting-glucose findings were preliminary because configural fit was weak. The self-management → distress → quality-of-life pathway showed no detected sex difference but should not be interpreted as equivalent across sex. Educational attainment moderated the pathway, with full mediation through distress only among tertiary-or-higher participants. LMIC diabetes research routinely compares subgroups using translated instruments; measurement equivalence testing should therefore be part of future PRO adaptation and validation pipelines.

## Figures and Tables

**Table 1:** Descriptive characteristics by sex group (*n* = 374).

Characteristic	Female (*n* = 152)	Male (*n* = 222)
Age, mean (SD), years	53.0 (2.9)	52.8 (2.8)
BMI, mean (SD), kg/m^2^	22.9 (2.2)	23.0 (2.1)
FBG, mean (SD), mg/dL	150.1 (24.9)	149.0 (24.3)
Diabetes duration, mean (SD), y	8.5 (3.0)	8.4 (2.9)
Lower-FBG (<154 mg/dL), *n*	84	128
Tertiary+ education, *n*	75	97

**Table 2:** McDonald’s ω per instrument and grouping variable.

Grouping	Group	Instrument	ω
Sex	Female	DSMI35	0.968
Sex	Female	DDS17	0.989
Sex	Female	AsianDQOL	0.986
Sex	Male	DSMI35	0.970
Sex	Male	DDS17	0.989
Sex	Male	AsianDQOL	0.985
Fasting glucose	Lower-FBG	DSMI35	0.966
Fasting glucose	Lower-FBG	DDS17	0.982
Fasting glucose	Lower-FBG	AsianDQOL	0.970
Fasting glucose	Higher-FBG	DSMI35	0.959
Fasting glucose	Higher-FBG	DDS17	0.990
Fasting glucose	Higher-FBG	AsianDQOL	0.984
Education	Secondary or lower	DSMI35	0.966
Education	Secondary or lower	DDS17	0.984
Education	Secondary or lower	AsianDQOL	0.979
Education	Tertiary or higher	DSMI35	0.972
Education	Tertiary or higher	DDS17	0.987
Education	Tertiary or higher	AsianDQOL	0.990

**Table 3:** Multi-group CFA measurement equivalence results: fit indices and ΔCFI/ΔRMSEA across groupings.

Grouping	Step	CFI	RMSEA	SRMR	ΔCFI	ΔRMSEA	Decision
Sex	Configural	0.976	0.080	0.026	—	—	Baseline
Sex	Metric	0.977	0.076	0.027	−0.0010	−0.0045	Supported
Sex	Scalar	0.974	0.079	0.035	0.0037	0.0032	Supported
Fasting glucose	Configural	0.943	0.119	0.041	—	—	Baseline
Fasting glucose	Metric	0.940	0.118	0.060	0.0036	−0.0006	Supported
Fasting glucose	Scalar	0.937	0.117	0.066	0.0030	−0.0011	Supported
Education	Configural	0.972	0.087	0.026	—	—	Baseline
Education	Metric	0.970	0.087	0.043	0.0020	−0.0001	Supported
Education	Scalar	0.970	0.084	0.044	0.0002	−0.0025	Supported

**Table 4:** Structural path coefficients and bootstrap indirect effects by group.

Grouping	Group	Effect	B [95% CI]
Sex	Female	Indirect	0.553 [0.199, 1.746]
Sex	Female	Direct	0.926 [0.251, 1.777]
Sex	Female	Total	1.479 [1.049, 2.476]
Sex	Male	Indirect	0.727 [0.343, 1.795]
Sex	Male	Direct	0.726 [0.265, 1.211]
Sex	Male	Total	1.453 [1.083, 2.241]
Education	≤Secondary	Indirect	0.308 [0.113, 0.790]
Education	≤Secondary	Direct	1.244 [0.779, 1.857]
Education	≤Secondary	Total	1.551 [1.216, 2.165]
Education	Tertiary+	Indirect	1.859 [1.022, 5.321]
Education	Tertiary+	Direct	0.157 [−0.152, 0.523]
Education	Tertiary+	Total	2.017 [1.240, 5.321]

## Data Availability

Data are available from the corresponding author on reasonable request.

## Supplementary Materials

The following supporting information can be downloaded at: https://www.mdpi.com/article/10.3390/nursrep16060205/s1, File S1, STROBE checklist (cross-sectional studies); File S2, bootstrap-distribution diagnostics (Tables S1A–S1C), percentile-versus-BCa sensitivity (Table S1D), and fasting-glucose latent factor diagnostics (Table S1E).

## Public Involvement Statement

No patients or members of the public were involved in the design, conduct, or reporting of this secondary analysis.

## Guidelines and Standards Statement

This study was conducted and reported in accordance with the STROBE (Strengthening the Reporting of Observational Studies in Epidemiology) guideline for cross-sectional studies [21]; the completed checklist is provided in Supplementary File S1.
