# Supplementary material for: Measurement Equivalence of Diabetes Self-Management, Distress, and Quality-of-Life Measures in Adults with Type 2 Diabetes in Vietnam
Source: Nurs Rep. 2026 Jun 18;16(6):205. doi: 10.3390/nursrep16060205 (PMC13306199; doi:10.3390/nursrep16060205)
Supplement: Supplementary file 1 [file nursrep-16-00205-s001.zip › Supplementary_File S1_STROBE_checklist.pdf]

Supplementary File S1 — STROBE Statement Checklist

Manuscript: Measurement equivalence of diabetes self-management, distress, and quality-of-life measures in adults with type 2 diabetes in Vietnam

Study type: Cross-sectional (secondary analysis)

STROBE version: STROBE Statement v4 (2007) — checklist for cross-sectional studies

Reference: von Elm E, et al. Lancet. 2007;370(9596):1453–1457. PMID: 18064739.

#	Item	Recommendation	Reported on page / section
Title and Abstract			
1a	Indicate the study's design with a commonly used term in the title or the abstract	Title: "Measurement equivalence ... in adults with type 2 diabetes in Vietnam" (study design — cross-sectional secondary analysis — appears in Abstract Methods and §2.1)	Title; Abstract Methods; §2.1
1b	Provide in the abstract an informative and balanced summary of what was done and what was found	Structured abstract with Background, Methods, Results, Conclusions	Abstract
Introduction			
2	Background/rationale: explain the scientific background and rationale for the investigation being reported	LMIC PRO comparison burden; equivalence as prerequisite; gap in Vietnamese T2DM	§1 ¶1–¶4
3	Objectives: state specific objectives, including any prespecified hypotheses	Primary: MG-CFA equivalence across sex, fasting glucose stratum, education. Secondary: structural path comparison where ≥ metric equivalence established.	§1 ¶5
Methods			
4	Study design: present key elements of study design early in the paper	"Secondary analysis of cross-sectional dissertation data"	§2.1

| 5 | Setting: describe the setting, locations, and relevant dates, including periods of recruitment, exposure, follow-up, and data collection | Two private hospitals, Binh Duong Province, Vietnam, June–July 2024 | §2.1 |

| 6a | Participants: give the eligibility criteria, and the sources and methods of selection of participants | Adults with confirmed T2DM, age ≥ 18 years, able to complete self-report questionnaires | §2.1 |

| 7 | Variables: clearly define all outcomes, exposures, predictors, potential confounders, and effect modifiers. Give diagnostic criteria, if applicable | DSMI-35 (5 subscales, 35 items); DDS-17 (4 subscales, 17 items); AsianDQOL (5 subscales, 21 items); grouping variables = sex, FBG stratum at 154 mg/dL, education tertiary-vs-≤secondary | §2.2, §2.3 |

| 8 | Data sources/measurement: for each variable of interest, give sources of data and details of methods of assessment (measurement). Describe comparability of assessment methods if there is more than one group | All three instruments self-administered in Vietnamese; FBG cut point applied to recorded fasting glucose; demographics from study records | §2.2, §2.3 |

| 9 | Bias: describe any efforts to address potential sources of bias | Configural-fit caveats explicit (§3.3, §4.5); STROBE-aligned reporting; pre-specified equivalence criteria (Cheung & Rensvold; Chen) | §2.4, §3.3, §4.5 |

| 10 | Study size: explain how the study size was arrived at | All 374 participants from the parent dissertation; minimum cell $n \geq 100$ confirmed for each grouping (sex 152/222; FBG 212/162; education 202/172) | §2.3 |

| 11 | Quantitative variables: explain how quantitative variables were handled in the analyses. If applicable, describe which groupings were chosen and why | FBG dichotomized at 154 mg/dL as sample-stratification device (not clinical control); education dichotomized at tertiary cut-off; subscale parcels used as indicators | §2.3, §2.4 |

| 12a | Statistical methods: describe all statistical methods, including those used to control for confounding | MG-CFA (lavaan, MLR); configural → metric → scalar sequence; $\Delta CFI \leq 0.010$ / $\Delta RMSEA \leq 0.015$ criteria; bootstrap percentile CI ($n = 5,000$; set.seed = 2024) for indirect effects; scaled Satorra-Bentler LRT for structural path equality | §2.4 |

| 12b | Describe any methods used to examine subgroups and interactions | Three-grouping MG-CFA; secondary structural path equality LRT per grouping | §2.4 |

| 12c | Explain how missing data were addressed | Complete-case analysis (no item-level missingness in parcel variables); $n = 374$ in all models | §2.3 |

| 12d | If applicable, describe analytical methods taking account of sampling strategy | Not applicable (no complex sampling) | — |

| 12e | Describe any sensitivity analyses | Bootstrap-distribution diagnostics for subgroup indirect effects, plus percentile-vs-bias-corrected-and-accelerated (BCa) sensitivity for the indirect effects (Supplementary Table S1, including new Table S1D) | §2.4, Supplementary Table S1 |

| Results | | | |

| 13a | Participants: report numbers of individuals at each stage of study | All 374 participants retained; subgroup n reported per Table 1 | §3.1, Table 1 |

| 13b | Give reasons for non-participation at each stage | Not applicable (secondary analysis) | — |

| 13c | Consider use of a flow diagram | Not applicable for secondary cross-sectional analysis | — |

| 14a | Descriptive data: give characteristics of study participants and information on exposures and potential confounders | Table 1 (age, sex, BMI, FBG, diabetes duration by group) | §3.1, Table 1 |

| 14b | Indicate number of participants with missing data for each variable of interest | No missingness on parcel variables; all 374 included | §2.3 |

| 15 | Outcome data: report numbers of outcome events or summary measures | Subscale ω values per group (Table 2); MG-CFA fit per step (Table 3) | §3.2, Tables 2–3 |

| 16a | Main results: give unadjusted estimates and, if applicable, confounder-adjusted estimates and their precision (e.g., 95% CI). Make clear which confounders were adjusted for and why they were included | Unstandardized B with 95% CI for structural paths (Table 4 Panel A); percentile bootstrap 95% CI for indirect effects (Table 4 Panel B); β_std reported alongside | §3.4, Table 4 (Panels A/B) |

| 16b | Report category boundaries when continuous variables were categorized | FBG cut at 154 mg/dL stated as sample-stratification device; education cut at tertiary-or-higher stated; rationale provided | §2.3 |

| 16c | If relevant, consider translating estimates of relative risk into absolute risk for a meaningful time period | Not applicable | — |

| 17 | Other analyses: report other analyses done — e.g., analyses of subgroups and interactions, and sensitivity analyses | Three groupings × three equivalence levels (Tables 3, 4); structural LRT per grouping; bootstrap/BCa sensitivity for indirect effects | §3.3, §3.4 |

| Discussion | | | |

| 18 | Key results: summarise key results with reference to study objectives | §4.1 (Main finding) | §4.1 |

| 19 | Limitations: discuss limitations of the study, taking into account sources of potential bias or imprecision. Discuss both direction and magnitude of any potential bias | Cross-sectional; single province; FBG vs HbA1c; higher-FBG convergence anomaly; coarse education categorization; parcel-level (not item-level) equivalence | §4.7 |

| 20 | Interpretation: give a cautious overall interpretation of results considering objectives, limitations, multiplicity of analyses, results from similar studies, and other relevant evidence | §4.1–§4.6 collectively; causal language softened throughout | §4.1–§4.6 |

| 21 | Generalisability: discuss the generalisability (external validity) of the study results | Limited to Vietnamese T2DM in similar private-hospital settings; multi-site replication recommended | §4.7, §4.8 |

| Other Information | | | |

| 22 | Funding: give the source of funding and the role of the funders for the present study and, if applicable, for the original study on which the present article is based | "No specific grant from any funding agency..." | Front matter (Funding statement) |

Author affirmation: All 22 STROBE items applicable to a cross-sectional secondary analysis have been addressed within the main manuscript at the locations indicated. Items not applicable (12d, 13b, 13c, 16c) are marked accordingly.

Prepared: 2026-05-29 (updated for concise academic revision)

Manuscript version at preparation: concise academic revision (01_Manuscript_concise_academic.docx, 2026-05-29)
