# Supplementary material for: Measurement Equivalence of Diabetes Self-Management, Distress, and Quality-of-Life Measures in Adults with Type 2 Diabetes in Vietnam
Source: Nurs Rep. 2026 Jun 18;16(6):205. doi: 10.3390/nursrep16060205 (PMC13306199; doi:10.3390/nursrep16060205)
Supplement: Supplementary file 1 [file nursrep-16-00205-s001.zip › Supplementary_File S2.pdf]

Supplementary Table S1 — Bootstrap-distribution diagnostics for subgroup indirect, direct, and total effects

Manuscript: Measurement equivalence of diabetes self-management, distress, and quality-of-life measures in adults with type 2 diabetes in Vietnam

Source CSV: data/processed/P3_bootstrap_diagnostics.csv

Generating script: P3/code/qc/P3_qc_bootstrap_diagnostics.R

Settings: set.seed(2024); 5,000 bootstrap replications per subgroup; ML estimator; std.lv = TRUE; within-group SEM (P3_multigroup_SEM.R model model_structural_boot)

Generated: 2026-05-25

Table S1A — Indirect effect (a × b)

Grouping	Group	Original B	Boot mean	Boot median	Boot SE	95% CI (percentile)	Half-width / SE	Skewness	Excess kurtosis	n admissible	CI excludes 0
Sex	Female	0.553	0.793	0.560	9.895	[0.199, 1.746]	0.078	70.52	4,982.4	5,000 / 5,000	✓
Sex	Male	0.727	0.819	0.734	0.380	[0.343, 1.795]	1.91	1.94	7.40	5,000 / 5,000	✓
Education	≤Secondary	0.308	0.348	0.312	0.185	[0.113, 0.790]	1.83	2.02	8.37	5,000 / 5,000	✓
Education	Tertiary+	1.859	3.883	1.910	28.720	[1.022, 5.321]	0.075	19.24	401.21	5,000 / 5,000	✓

Table S1B — Direct effect (c′)

Grouping	Group	Original B	Boot mean	Boot SE	95% CI (percentile)	Half-width / SE	Skewness	CI excludes 0

Sex	Female	0.926	0.951	0.638	[0.251, 1.777]	1.20	37.55	✓
Sex	Male	0.726	0.717	0.232	[0.265, 1.211]	2.04	0.27	✓
Education	≤Secondary	1.244	1.266	0.274	[0.779, 1.857]	1.96	0.47	✓
Education	Tertiary+	0.157	0.137	0.555	[-0.152, 0.523]	0.61	-7.35	✗

Table S1C — Total effect (a×b + c′)

Grouping	Group	Original B	Boot mean	Boot SE	95% CI (percentile)	Half-width / SE	Skewness	CI excludes 0
Sex	Female	1.479	1.745	10.409	[1.049, 2.476]	0.069	70.57	✓
Sex	Male	1.453	1.537	0.300	[1.083, 2.241]	1.93	1.56	✓
Education	≤Secondary	1.551	1.615	0.242	[1.216, 2.165]	1.96	0.74	✓
Education	Tertiary+	2.017	4.020	28.597	[1.240, 5.321]	0.071	19.15	✓

Table S1D — Percentile vs bias-corrected-and-accelerated (BCa) sensitivity for the indirect effect

Sensitivity analysis (revision). Percentile CIs reproduce Table S1A / P3_indirect_{sex,education}.csv exactly; BCa CIs (bca.simple) are provided to show robustness of the zero-exclusion inference under heavy skew. Source CSV: data/processed/P3_indirect_bca_sensitivity.csv; generating script P3/code/qc/P3_bca_sensitivity.R (set.seed(2024); 5,000 replications; ML estimator).

Grouping	Group	Indirect B	95% CI (percentile)	95% CI (BCa)	BCa excludes 0
Sex	Female	0.553	[0.199, 1.746]	[0.195, 1.709]	✓
Sex	Male	0.727	[0.343, 1.795]	[0.333, 1.736]	✓

Education	≤Secondary	0.308	[0.113, 0.790]	[0.109, 0.766]	✓
Education	Tertiary+	1.859	[1.022, 5.321]	[0.984, 4.860]	✓

The BCa interval excluded zero for the indirect effect in all four displayed subgroups, agreeing with the percentile inference reported in the manuscript. For completeness, the direct and total effects are in the same source CSV: the only effect excluding zero under neither method is the Tertiary+ direct effect (percentile [−0.152, 0.523]; BCa [−0.134, 0.570]), consistent with the full-mediation pattern reported for that subgroup. The glycemic-stratum grouping is excluded from this sensitivity analysis because its higher-FBG within-group model was non-positive-definite and therefore not interpretable.

Table S1E. Fasting-glucose latent factor diagnostics (single-group confirmatory factor analysis, MLR).

Subgroup	n	Var(SM)	Var(DDS)	Var(QoL)	r(SM,DDS)	r(SM,QoL)	r(DDS,QoL)	Min eigenvalue	Non-positive-definite?
Lower-FBG (<154 mg/dL)	212	0.143	0.357	7.343	-0.603	0.606	-1.000	-0.00004	Yes
Higher-FBG (>=154 mg/dL)	162	0.080	0.664	10.085	-0.434	0.725	-0.557	+0.037	No

Note. Latent factor correlations and variances from single-group confirmatory factor models (MLR estimator; SM = self-management, DDS = diabetes distress, QoL = quality of life). In the lower-FBG subgroup the distress-quality-of-life latent correlation reached the -1.0 boundary, producing a non-positive-definite latent covariance matrix (smallest eigenvalue < 0) and precluding interpretation of the fasting-glucose structural comparison; the higher-FBG model was positive-definite. Source: P3_glycemic_heywood_diagnostics.csv (script P3/code/qc/P3_heywood_diagnostics.R, seed 2024).

Interpretation

Heavy-tailed subgroups (boldface ratios): Female (sex) and Tertiary+ (education) bootstrap distributions for indirect and total effects show half-width/SE ratios near 0.07–0.08, far from the ~1.96 expected for a symmetric Gaussian. Sample skewness in those distributions is 19–71, and

kurtosis is 400–5,000. This pattern arises from a small fraction of bootstrap replicates near local model non-identification producing very large absolute estimates that inflate the standard error without shifting the bulk of the distribution.

Approximately symmetric subgroups: Male (sex) and ≤Secondary (education) indirect distributions have ratios 1.83–1.91 and skewness 1.9–2.0 — close enough to Gaussian behaviour that either Wald or percentile inference would agree.

Inference rule (manuscript §2.4): Percentile bootstrap CIs are reported as primary inference in Table 4B and in body-text indirect-effect statements. The asymptotic z-test (Wald) p-values shown in the underlying CSV (P3_indirect_*.csv) are not used in the manuscript for the heavy-tailed subgroups because they are misleading in the presence of this degree of skew. The percentile choice was pre-specified prior to analysis; Table S1D provides BCa intervals as a sensitivity check, and the zero-exclusion inference is unchanged under BCa for all four displayed subgroup indirect effects.

Admissibility: lavaan flagged 0 of 5,000 replicates as outright non-admissible in any subgroup (all n_boot_admissible = 5,000). The "small fraction near local non-identification" is therefore a tail-extremity issue rather than a convergence failure — the affected replicates are admissible but produce far-from-typical estimates.

lavaan diagnostic notes from the run: lavaan emitted standard "high (>5) ratio of standard deviation to median absolute deviation" warnings for the parameter b in heavy-tailed subgroups and for the defined parameters indirect and total. These warnings are consistent with — and the proximate cause of — the half-width/SE divergence reported above; they do not indicate computational error.

Generated: 2026-05-25 by P3/code/qc/P3_qc_bootstrap_diagnostics.R (15 minute runtime on Apple Silicon). Reproduced exactly with set.seed(2024).
